# New Improvements in Pediatric Disease Diagnosis and Treatment

**DOI:** 10.3390/diagnostics16040542

**Published:** 2026-02-12

**Authors:** Elena Țarcă

**Affiliations:** Faculty of Medicine, Grigore T. Popa University of Medicine and Pharmacy, 700115 Iaşi, Romania; tarca.elena@umfiasi.ro

Pediatric patients can present significant diagnostic and therapeutic challenges, as their pathophysiology differs substantially from that of adults. The pediatric population encompasses a vast spectrum, ranging from premature newborns to adolescents up to eighteen years of age and from extremely low-birth-weight infants to patients weighing up to one hundred kilograms. This wide variability underscores the complexity of pediatric care. Although substantial progress has been made in understanding the biological mechanisms and management of several congenital and acquired pediatric diseases, such as mucoviscidosis, Niemann–Pick disease, and celiac disease [1,2,3], outcomes for other conditions remain suboptimal. These include genetic and metabolic disorders, severe congenital malformations, and neonatal sepsis, highlighting the ongoing need for research and innovation in pediatric medicine [4].

This Special Issue includes original research articles, reviews, and case reports that collectively highlight the breadth and depth of contemporary pediatric diagnostic research, addressing several important key topics. Chromosomal anomalies, extremely low birth weight, and prematurity are frequently associated with congenital deformities, representing a broad and clinically significant area of study. Accurate syndromic classification, supported by imaging, genetic testing, and clinical correlation, remains essential for guiding management and counseling families [5]. This is particularly relevant in cases of anorectal malformations and congenital anterior abdominal wall defects, which are often associated with cardiac or pulmonary anomalies, further complicating surgical management and worsening prognosis [6,7,8,9]. Surgical techniques also must be tailored not only to the patient’s age and weight but to specific requirements related to anesthesia, medication dosing, and blood transfusions [10,11].

Another important pillar of this Special Issue is the development of genetic analysis and molecular diagnostics. The increasing availability of next-generation sequencing, improved cytogenetic techniques, and refined genotype–phenotype correlations has transformed the diagnostic landscape for inherited and metabolic diseases [1]. Despite these advances, many disorders continue to have limited therapeutic options, underscoring the importance of early diagnosis, supportive care optimization, and ongoing translational research. Neonatal intensive care, pediatric oncology and gastroenterology similarly present challenges distinct from those encountered in adult populations, requiring specialized approaches to care [12]. Advances in medical sciences and technology have enabled increasingly individualized treatment strategies, allowing clinicians to tailor interventions to achieve optimal outcomes.

The breadth of research topics addressed in this Special Issue on pediatric pathology, as outlined in the Contributions list, reflects the comprehensive nature of pediatric medicine. The studies included employ a range of methodological approaches, including systematic and literature reviews, as well as prospective and observational designs. The findings yield significant insights that can be extrapolated beyond the original study contexts, and their dissemination contributes to the advancement of the scientific and medical community. Notably, some articles and case reports address practical challenges commonly encountered in clinical practice, providing readers with valuable insights into current research on genetic, congenital, neonatal, and pediatric pathology, as well as their application to routine clinical care [13,14,15].

Conclusions

Congenital abnormalities, maternal–fetal medicine, and genetics are among the most rapidly advancing fields in modern medicine. Technological innovations, novel imaging techniques, and the development of new therapeutic strategies have significantly improved our understanding and management of neonatal and pediatric conditions. Considering patient age, weight, anatomy, disease biology, and developmental stage, predictive and preventive medicine, as well as personalized medical approaches, are becoming increasingly integral to pediatric diagnosis and treatment.

Aiming to improve clinical outcomes in pediatric populations, this Special Issue highlights the complexity of pediatric and congenital disorders and underscores the importance of multimodal diagnostic approaches that integrate clinical evaluation with advanced imaging techniques, laboratory biomarkers, and histopathological assessment. It further emphasizes the value of individualized therapeutic strategies and multidisciplinary, translational research.

Future innovations in wearable technology, regenerative medicine, genomics, and artificial intelligence have the potential to improve long-term outcomes, early diagnosis, and treatment in children, particularly in rare, genetic, and severe congenital conditions.
